# Delayed-Type Hypersensitivity to *Mycobacterium tuberculosis* Antigens: The Immunological Mechanism and Potential Therapeutic Strategies—A Systematic Review

**DOI:** 10.3390/ijms27062620

**Published:** 2026-03-13

**Authors:** Wiktoria Andryszkiewicz, Maksym Bodziony, Milena Chmielewska, Martyna Kowalczyk, Gabriela Rzońca, Krzysztof Gomułka

**Affiliations:** 1 Student Research Group of Allergology and Internal Medicine, Faculty of Medicine, Wroclaw Medical University, 50-556 Wrocław, Poland; wiktoria.andryszkiewicz@student.umw.edu.pl (W.A.); maksym.bodziony@student.umw.edu.pl (M.B.); milena.chmielewska@student.umw.edu.pl (M.C.); martyna.kowalczyk@student.umw.edu.pl (M.K.); gabriela.rzonca@student.umw.edu.pl (G.R.); 2 Clinical Department of Allergology and Internal Medicine, Institute of Internal Medicine, Wroclaw Medical University, 50-556 Wrocław, Poland

**Keywords:** delayed-type hypersensitivity, *Mycobacterium tuberculosis*, mantoux test, tuberculosis vaccines, purified protein derivative

## Abstract

Delayed-type hypersensitivity (DTH) to *Mycobacterium tuberculosis* (MTb) antigens is a crucial component of the cellular immune response presented during tuberculosis infection. This reaction is driven primarily by T lymphocytes, which recognize mycobacterial antigens and trigger a focused inflammatory cascade. Cytokines produced by T lymphocytes stimulate the formation of granulomas, organized structures that help contain the bacteria and prevent their spread. DTH is essential for controlling the infection and forms the basis of diagnostic tools, including the still widely practiced tuberculin skin test despite its limitations. This immunological mechanism is also used as an important therapeutic target in the treatment of tuberculosis by modulating the cellular response. These approaches include immunomodulatory agents, therapeutic vaccines and host-directed treatment. Ongoing research offers promising opportunities for future interventions aimed at decreasing the global mortality associated with tuberculosis.

## 1. Introduction

*Mycobacterium tuberculosis* (MTb) is an aerobic bacillus belonging to the *Mycobacteriaceae* family of the *Actinobacteria* order [1]. In 1882, Robert Koch, a German microbiologist, described the successful isolation of the causative agent of tuberculosis, which was subsequently named MTb a year later [2]. MTb is the bacterium that causes tuberculosis, the second deadliest infectious disease, and is particularly deadly among people living in lower-income countries where Human Immunodeficiency Virus (HIV) prevalence is high. Tuberculosis has claimed over 1.3 million deaths worldwide [3].

The primary route of transmission for MTb is airborne. MTb can be acquired through inhalation of tiny droplet nuclei, 1–5 µm in diameter, with several bacilli. Mechanisms such as coughing, sneezing, talking, laughing, singing, or normal breathing can produce droplet nuclei, which can then reach the alveoli in the lungs [4]. There are many factors that differentially influence susceptibility to MTb infection, demonstrating the complex interaction of multiple elements that determine the transmission of MTb. Transmission of tuberculosis depends not only on pathogenic and environmental factors but also on host characteristics such as age, sex, smoking, Bacillus Calmette–Guérin (BCG) vaccination status, comorbidities, previous tuberculosis, the presence of pulmonary cavities, and the severity and duration of symptoms [5].

MTb possesses inherent resistance mechanisms, including a thick, hydrophobic cell wall and drug-modifying enzymes, and additional resistance develops because of chromosomal mutations that affect drug targets or prodrug activation [6]. Moreover, another component that MTb owes its effectiveness as a pathogen and its natural resistance to numerous antimicrobial drugs is efflux pumps that can remove antibiotics from the bacteria, reducing their intracellular concentration and effectiveness [7]. In MTb infections, the importance of other diseases, the presence of which predisposes to tuberculosis, should also be emphasized. A 2024 scientific article conducted a meta-analysis that included 48 retrospective and prospective cohort studies with over 61 million participants from six World Health Organization (WHO) regions. Eight population-based studies were from South Korea, 19 from China, with overlapping study periods, and only one from the African region, Ethiopia. The aim of this study was to determine the prognostic value of diabetes in the general population of adults, adolescents, and children in predicting tuberculosis disease. The conclusions indicated that diabetes likely increases the risk of developing tuberculosis in both the short- (<10 years) and long-term (≥10 years). Glycemic control and access to healthcare are important factors, as these factors can modify this risk [8]. Another meta-analysis conducted in 2024, which included 51 cohort studies with over 27 million participants from six WHO regions, also highlighted the role of malnutrition as a contributing factor to the development of tuberculosis [9]. Furthermore, a 2025 meta-analysis found that the prevalence of pulmonary tuberculosis among adults with HIV or acquired immunodeficiency syndrome (AIDS) in Ethiopia was 15%, significantly higher than the estimated prevalence in the general HIV-negative population, which is less than 1%. The risk was particularly increased in individuals with low CD4^+^ T-cell counts, advanced HIV infection, and tobacco use. These findings underscore the need for early detection, targeted interventions, and integration of tuberculosis control programs with HIV care [10,11].

Host defense mechanisms include both innate and adaptive immune responses. Elements of the innate immune response include the respiratory mucosa. It consists of elements such as the epithelium, the airway epithelial cell layer (AEC), which forms a barrier against invasion; the lamina propria, the connective tissue layer; immune cells, including lymphocytes and macrophages; the airway surface fluid (ASL); and a complex substance containing mucus, immunoglobulin A, and various other innate immune factors on the surface of the airway lumen. Respiratory epithelial cells recognize MTb via molecular pattern receptors, activate MAIT cells, and secrete antibacterial peptides and cytokines, supporting early innate immunity and macrophage activation. Dendritic cells (DCs) are another component of the innate immune response. They recognize MTb, phagocytize it, and then present antigens to T lymphocytes, thereby linking innate and adaptive immunity, although MTb can replicate in DCs and partially disrupt their function. Neutrophils in tuberculosis play a complex role because they can both limit and promote the growth of MTb. Neutrophils also secrete cytokines and chemokines that recruit other immune cells, and their granule content may contribute to supporting bacterial killing in macrophages. Cells such as natural killer T (NKT) and γδ T lymphocytes participate in the early response to MTb by activating macrophages via IFN-γ, destroying infected cells, and supporting the development of a T-cell response [12].

The body fights against the pathogen to prevent it from developing an infection. Hypersensitivity is an excessive or pathogenic immune response, which Gell and Coombs classified into four types, and contemporary research is modifying this classification, describing the immunological mechanisms of each type [13]. The original Gell and Coombs classification divides hypersensitivity reactions into four subtypes depending on the type of immune response and the effector mechanism responsible for cell and tissue damage: type I—immediate or immunoglobulin E (IgE)-dependent; type II—cytotoxic or IgG/IgM-dependent; type III—IgG/IgM immune complex-dependent; and type IV—delayed or T-cell-dependent hypersensitivity [14]. DTH is a delayed-type reaction and involves two phases of sensitization: primary immunization with the specific antigen and the second phase, the effector DTH response, which typically occurs 6–14 days after sensitization [15].

Modern immunological research has refined the original classification of hypersensitivity proposed by Gell and Coombs, dividing type IV hypersensitivity into distinct subcategories: type IVa (dependent on Th1 cells and macrophage activation), type IVb (dependent on Th2 cells and eosinophilic inflammation), type IVc (mediated by cytotoxic CD8^+^ T cells), and type IVd (dependent on T cells and neutrophilic inflammation). This expanded classification now accounts for both distinct immunopathological mechanisms and clinical variations based on the predominant immune cells involved, extending beyond the classical T-cell-mediated response [14,16].

The latest update of the WHO 2025 recommendations is now available, covering the diagnosis of tuberculosis, active disease, and drug resistance [17]. The new guidelines cover two classes of molecular Nucleic Acid Amplification Tests (NAATs): the low-complexity automated nucleic acid amplification test (LC-aNAAT) and low-complexity manual nucleic acid amplification (LC-mNAAT). The LC-aNAAT and LC-mNAAT are low-complexity tests intended for use outside specialized reference laboratories. LC-aNAATs are automated tests that are performed with minimal operator intervention, and the examples are Xpert MTB/RIF, Xpert Ultra, and Truenat. LC-mNAATs are manual tests that require more manual steps and often require visual interpretation. Examples of such tests include TB-LAMP (Tuberculosis Loop-mediated Isothermal Amplification). TB-LAMP is a manual NAAT method based on isothermal DNA amplification. Results can be read visually relatively quickly under ultraviolet light. The WHO recommends TB-LAMP as an alternative to microscopy in the diagnosis of pulmonary tuberculosis. These tests are becoming the basis for first-line diagnostics in suspected tuberculosis, replacing microscopy and culture. A significant change concerns the simultaneous testing of respiratory and extrapulmonary samples in patients at increased risk of severe MTb infection, particularly in people living with HIV, as well as in children [17,18,19].

For symptomatic adults and adolescents with a positive respiratory specimen, NAAT testing is recommended as the first-line test. It is worth mentioning that NAAT testing has also become a method for detecting resistance to rifampicin and, in some situations, to other drugs such as isoniazid, fluoroquinolones, amikacin, and ethionamide. The role of NAAT testing in the latest guidelines has been expanded to include extrapulmonary tuberculosis and tuberculous meningitis, where these tests on appropriate fluids and tissues are recommended as first-line tests. The new guidelines also mention interferon gamma release assay (IGRA) tests, which, according to the update, are used only to detect latent infection and not to diagnose active tuberculosis. All actions undertaken are aimed at improving the detection of extrapulmonary and drug-resistant forms and more effective global tuberculosis control [20].

## 2. Materials and Methods

### 2.1. Search Strategy

A comprehensive search using the PubMed database was conducted to recognize studies on DTH responses to *M. tuberculosis* antigens, using keywords such as “delayed-type hypersensitivity,” “tuberculosis,” and “immunomodulation”, “mantoux test”, “tuberculosis vaccines”.

### 2.2. Inclusion and Exclusion Criteria

Eligibility for inclusion was determined according to the following criteria: (1) publication in a peer-reviewed journal and (2) alignment with the review topic. Being written in the English language was preferable, although one study written in Spanish was included.

Exclusion criteria included: (1) lack of relevance to the review topic, (2) insufficient contribution to the objectives of the review and (3) duplicate records.

### 2.3. Literature Selection

**Figure 1:**
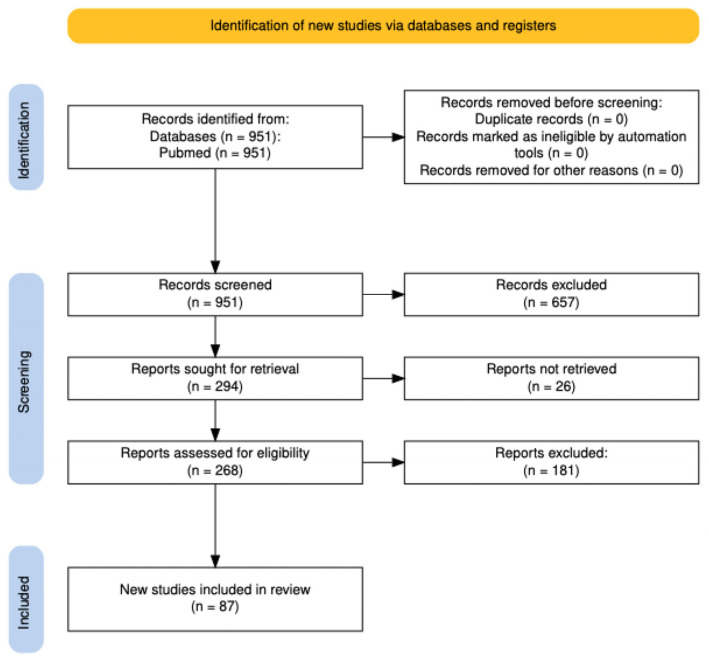
PRISMA flow diagram presenting study selection process.

The literature selection prioritized recent publications, particularly those from 2021 onwards, and was limited to articles published in English. An initial screening of titles and abstracts were reviewed to determine relevance, and full-text articles meeting eligibility criteria were subsequently appraised for methodological rigor and scientific soundness. A total of 87 studies were retained for the final analysis [Figure 1].

## 3. Results

### 3.1. Immunopathogenic Mechanism of Delayed-Type Hypersensitivity (DTH) in MTb Infection

The tuberculosis bacillus, after entering the lungs, is quickly absorbed by alveolar macrophages and DCs. Its recognition involves numerous innate immunity receptors, including Toll-like receptors (TLR)—TLR-2, TLR-4, and TLR-9, as well as C-type lectin receptors (for example, DC-SIGN, MARCO), various scavenger receptors, and complement receptors (CR3/CR4). Through them, phagocytic cells not only initiate phagocytosis but also activate their typical signaling pathways, which results in the release of pro-inflammatory cytokines and chemokines. This stage forms the basis for the subsequent activation of type 1 T helper (Th1) lymphocytes [21,22,23,24].

However, MTb has a set of mechanisms that allow it to weaken the effectiveness of this early response. The best-known include factors that inhibit phagosome maturation, such as SapM, PtpA, and PknG, as well as ESX-1 secretion system proteins that disrupt the integrity of the phagosome membrane. Maintaining the phagosome in an immature state shields the pathogen from degradation and allows it to persist inside host cells [21,22,25].

Following antigen processing and its display on MHC class II molecules by dendritic cells in regional lymph nodes, naïve CD4 T-cells undergo activation. In an environment rich in interleukin 12 (IL-12) and IL-18, these cells differentiate into Th1 lymphocytes. Their key product is interferon-γ (INF-γ), which is the main signal that activates macrophages and conditions their ability to control MTb growth. Tumor necrosis factor α (TNF-α), which is responsible for preserving granuloma integrity, is equally important [21,26]

The DTH response in tuberculosis usually develops several weeks after infection and relies heavily on the activity of Th1 lymphocytes. Upon re-exposure to MTb antigen in the lungs, IFN-γ and TNF-α are secreted, and chemokines recruit additional immune cells. Under the influence of IFN-γ, macrophages enhance their generation of reactive oxygen and nitrogen species and shift to a more pro-inflammatory phenotype [21,27,28].

As the infection progresses, an organized granulomatous structure forms, in which the center is an area of necrosis surrounded by various populations of cells: macrophages, epithelial cells, giant cells, and lymphocytes. The seed-like structure limits the spatial infection, although the bacterium itself can survive in a dormant state within it. Its maintenance depends primarily on IFN-γ and TNF-α, which determine macrophage activity [21,29,30].

High levels of nitric oxide (NO) and reactive oxygen species (ROS) are generated inside the granuloma [28]. These molecules have a bactericidal effect, but their excessive production can lead to damage to the host cells and promote the formation of caseous necrosis. As the process progresses, the granuloma structure weakens, which can lead to its disintegration. The situation is particularly unfavorable when an excessive number of neutrophils participate in the reaction; their enzymes and generated free radicals intensify tissue destruction and promote the formation of cavities. Although TNF-α is necessary for maintaining the proper structure of the granuloma, its excessive production can contribute to lung damage and promote cavitation. In such an environment, the bacilli have a greater chance of moving into the air spaces and further transmission [21,29,30].

Some components of the MTb wall, such as phenolic lipids, can further modulate the immune response by weakening cytokine production [19]. Excessive recruitment of monocytes or neutrophils, dependent on CC chemokine receptor 2 (CCR2) and IL-17 signaling, can disturb immune homeostasis and drive the development of a chronic inflammatory state that promotes tissue destruction. As a result, immunity against MTb results from a continuous balance between protective mechanisms and processes that, if they get out of control, lead to damage of the host tissues [21,31].

#### 3.1.1. Role of Cytokines in Hypersensitivity in MTb

In patients with active pulmonary tuberculosis, elevated levels of both IL-10 and transforming growth factor-β (TGF-β) have been reported in the lungs. However, mixed results among different experimental models have been described. One study showed that IL-10-deficient C57BL/6 mice exhibited higher bacterial loads in the pulmonary tissue and increased mortality in later stages of infection. In contrast, other studies showed IL-10-deficient C57BL/6 and BALB/C mice have reduced bacterial burdens in their lungs later during the infection [32,33]. Additionally, treatment of CBA/J mice with an anti-IL-10 receptor-blocking antibody during the chronic stage of MTb helps to lower the number of bacteria in the lungs and to improve survival rates [34]. These discrepancies across different experimental models may reflect the differences among the studied populations.

TGF-β is a cytokine of fundamental value to the balance of the immune response in MTb infections. This molecule cytokine has an inhibitory effect on cells involved in the regulation of the immune mechanisms. These include: macrophages, neutrophils, DCs, and T cells [35]. TGF-β concentrations correspond with the severity of this disease [36]. Blocking TGF-β signaling enables the organism to increase the control of the disease by lowering the number of bacteria in the pulmonary tissue. This effect can be achieved by an antibody, recombinant TGF-β receptor or an inhibitor [37]. That suggests a suppressive immune response to MTb. One study reported the possibility of limiting effective macrophage activation through TGF-β by preventing CD4 T-cells’ production of IFN-γ in the centers of granulomas [21,38].

Immunity to MTb and suppression of bacterial replication are possible due to adaptive immune responses. Cytokine production and direct effector mechanisms of antigen-specific T cells are essential to the adaptive immunity in this infection. Upon exposure to MTb, both effector and memory cells are produced. The latter enables scientists to develop vaccines that induce immunity against microbes. Upon re-exposure these cells enable a rapid immune response due to the long-lasting activity of the memory cells. Nevertheless, adaptive immune responses can sometimes be harmful either by triggering exaggerated inflammation or becoming unsuccessful from prolonged antigen exposure. CD4 T-cell responses during MTb infection are of great importance. As reviewed, these cells activate macrophages via IFN-γ, they control bacterial replication, maintain the structure of the granuloma, generate immunological memory and are a part of the regulatory mechanisms to prevent excessive damage [21]. The DTH mediators and their role in tuberculosis are presented in Table 1.

**Figure 2:**
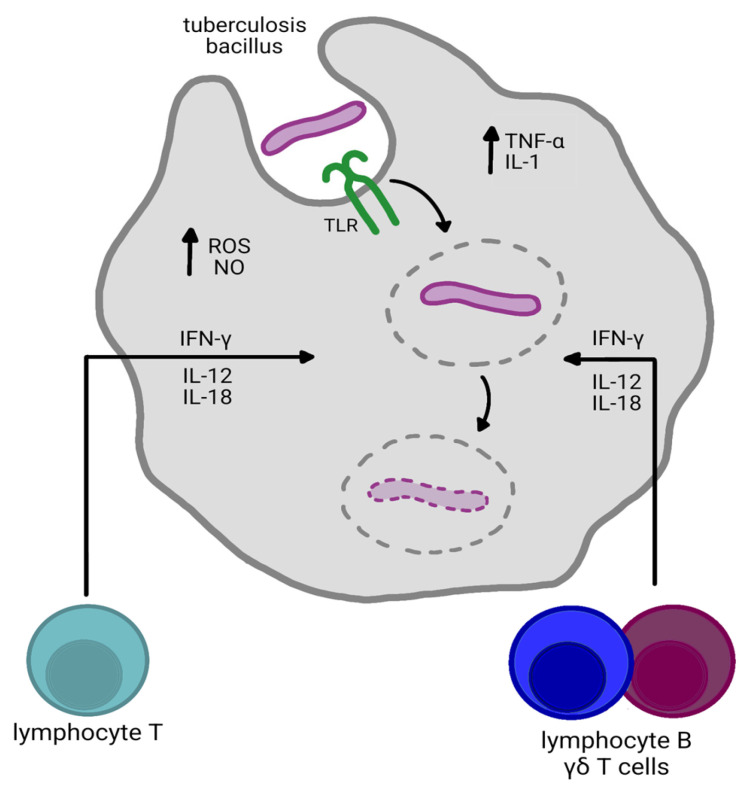
Cytokines and delayed-type hypersensitivity in tuberculosis. IFN-γ—macrophage activation; IL-12, IL-18—stimulation; IL-1, TNF—promote cytokine production; ROS, NO—induction of effector molecules.

Detectable memory T-cell responses have been observed in patients with latent tuberculosis infection (LTBI) and active tuberculosis after successful treatment [46]. Memory T cells have been studied in mouse models that, after MTb infection, received antibiotic therapy and then were reinfected. Memory T cells were abruptly recruited to fight the infection. Memory CD4 T cells respond more rapidly than naive T cells, indicating that memory enables enhanced efficiency of immune response during reinfection. It is due to the higher precursor frequency of memory cells. Though comparing the timing of the start of division, both naive and memory T-cells have a similar delay (2–3 days) [47,48]. Figure 2 illustrates the cytokines mentioned in our study that are involved in the development of hypersensitivity reactions.

#### 3.1.2. Immune Response Balance in Tuberculosis

Homeostasis of the immune response during an MTb infection is crucial to avoid the damage caused by the response itself. Such regulation of the immune system is possible via regulatory T cells (Treg) that suppress certain response mechanisms. Low levels of Treg cells can cause autoimmunity, and conversely, high levels cause immunosuppression [49]. Treg cell count increase is found in active tuberculosis but not in latent tuberculosis. Moreover, both CD28 and CD8 T cells tend to have much lower counts in active tuberculosis compared to the LTBI [50]. Sustained expansion of Treg cells has been associated with MDR tuberculosis. These cells appear to mobilize the body’s effector immune responses, and blocking the PD-1 pathway on these cells greatly improved protective T-cell activity. This indicates that the pathway plays an important role in restoring immune function in people with tuberculosis. The Treg-driven suppression of protective immunity relied on the programmed cell death protein 1 (PD-1) and its ligand (PD-L1) interaction, and Tregs remained abundant in patients with multidrug-resistant tuberculosis who failed to respond to conventional chemotherapy [43].

Data collected from animal-based studies suggest that after successful treatment of tuberculosis with standard drugs, the memory T-cell response is not long-lasting because of exhaustion. This was based on a study on mice infected with tuberculosis after chemotherapy and reinfection, where a Treg cell response was not noticeable. T cells collected from lung tissue showed evidence of PD-1 expression [51].

### 3.2. Hypersensitivity to MTb Antigens in the Diagnosis

As tuberculosis is responsible for the most deaths in the world, proper testing is crucial in the prevention of this disease and limiting its spread. The Mantoux test (MT) is based on DTH, involving administration of purified protein derivative (PPD) via the intradermal route derived from cultures of MTb, and it is still used to detect active and latent infection. The test is routinely employed to assess immune responsiveness to tuberculosis in individuals of all ages. A positive reaction occurs in patients who have previously had contact with MTb antigens or those who have been vaccinated with the BCG vaccine [52,53]. The MT is performed by intradermal injection of a typical dose of five tuberculin units (0.1 mL) and the results should be read between 48 and 72 h after the injection. The area of induration should be measured in millimeters (mm). As was said before, the MT represents DTH of skin and is caused by macrophages, monocytes and T cells. The lymphokines produced by T cells cause skin induration by widening local blood vessels, causing swelling, making fibrin build up, and attracting more inflammatory cells to the area [54]. The test should be interpreted by determination of the width of the localized hardening. The width of the localized hardening being 0–4 mm is considered negative, ≥5 mm is classified as positive in high-risk individuals, ≥10 mm in those at moderate risk, and ≥15 mm in individuals without tuberculosis risk factors However, the studies showed that the size does not matter, and the test has a low likelihood of correctly identifying true cases [55,56]. The interpretation of the MT is summarized in Table 2 [57].

Although the MT is still commonly used, it can be affected by many factors and result in false-positive and false-negative results. It is important to properly perform the test, as repeated testing leads to stronger tuberculin reactions, a lower dose of PPD may cause a false-negative result and a higher dose may cause a false-positive result. Moreover, infection with non-tuberculous mycobacteria and BCG vaccination can also lead to false-positive results; however, the U.S. advises that people who received the BCG vaccine can still get an MT and the fact that they were vaccinated should not change how the test results are read. Therefore, the interpretation of ≤18 mm among BCG-vaccinated people under the age of 40 should be done in a careful manner, especially if the patient is at low risk of infection, as the previous BCG vaccination can cause a positive result and not the MTb infection itself [53,58,59]. MT may incorrectly indicate a negative result among patients with T-cell deficiency, such as HIV-positive patients. The limitation of the test comes from the varied prevalence of tuberculin reactivity caused by different absolute CD4 lymphocyte counts and, therefore, should be interpreted cautiously, as the result of the test depends on the immune status [60]. However, even though MT is not the ideal method of screening, due to its simplicity, in some cases it is the only available method for TB screening, especially in developing countries [61].

The limitations of the MT were the inspiration for IGRAs such as QuantiFERON (QIFN). This test measures IFN-γ, which is secreted by T lymphocytes following in vitro exposure of leukocytes with PPD, using an ELISA method [62]. IGRA tests have multiple undeniable advantages over MT. The test can be read after 24 h and does not require a second visit, while in the MT, the second visit is needed for the induration measure and interpretation. Automated reading minimizes the influence of human interpretation bias. QIFN does not expose patients to antigen, so it prevents the anamnestic response, and therefore, repeated testing does not affect results. Additionally, QIFN is not influenced by prior BCG vaccination and is expected to be minimally affected by previous exposure to non-tuberculous mycobacteria. What is more, IGRAs demonstrate significantly higher accuracy in diagnosing latent tuberculosis compared with the MT because fewer and more-specific antigens are used in QIFN. Their major disadvantage is high cost [58,63].

## 4. Discussion

### 4.1. Modulation of the Immune Response in Tuberculosis—Potential Therapeutic Approaches

#### 4.1.1. Immunomodulation as an Adjunctive Strategy in Tuberculosis Treatment

The current standard of tuberculosis therapy is based on antimicrobial agents that directly target MTb. Standard anti-tuberculosis treatment regimens require prolonged administration and are frequently associated with significant adverse events. In drug-susceptible tuberculosis, the recommended first-line regimen includes isoniazid (INH), rifampicin (RMP), pyrazinamide (PZA), and ethambutol (EMB) [64]. In multidrug-resistant tuberculosis (MDR-TB), therapeutic options include fluoroquinolones (moxifloxacin, levofloxacin), linezolid, clofazimine, and meropenem, as well as next-generation agents such as bedaquiline, pretomanid and delamanid [65].

The escalating global burden of antimicrobial resistance has intensified the search for novel therapeutic strategies. Host-directed therapy (HDT) has emerged as a promising strategy that aims to modulate the host immune response rather than directly targeting the pathogen [66]. The fundamental aim of HDT in tuberculosis treatment is twofold: to enhance antimicrobial host mechanisms while simultaneously limiting excessive inflammation and tissue-destructive immunopathology [67].

#### 4.1.2. Vitamin D

Vitamin D plays a pivotal role in the modulation of innate immune response against intracellular pathogens [68]. It induces the expression of antimicrobial peptides, particularly cathelicidin (LL-37)—an antimicrobial peptide that concurrently induces MTb death and modulates host immune responses by downregulating pro-inflammatory mediator production—which promotes autophagy in macrophages, thereby restricting intracellular MTb replication. In small clinical trials, supplementation in vitamin D-deficient individuals has been shown to significantly enhance IFN-γ secretion following stimulation with MTb antigens, correlating with accelerated clinical and radiological improvement in pulmonary tuberculosis. However, outcomes in larger randomized controlled trials evaluating adjunctive vitamin D supplementation in immunocompetent patients with normal baseline vitamin D levels remain inconsistent and inconclusive [69,70,71].

#### 4.1.3. Phenylbutyrate and Loperamide

Phenylbutyrate (PBA), a histone deacetylase inhibitor, exerts immunomodulatory effects comparable to vitamin D by upregulating cathelicidin (LL-37) expression and promoting autophagolysosome formation, thereby accelerating intracellular MTb degradation. Synergistic activity between vitamin D and PBA has been demonstrated in vitro in inhibiting MDR-TB replication in macrophages and in enhancing the antimicrobial efficacy of RMP and INH [72]. Loperamide, a peripherally acting synthetic opioid, has also been demonstrated in vitro to induce autophagy and upregulate LL-37 expression in MTb-infected macrophages [71].

#### 4.1.4. Rapamycin

Rapamycin, a potent inhibitor of the mammalian target of rapamycin (mTOR) kinase, has received increasing attention in tuberculosis research due to the pivotal role of the mTOR pathway in suppressing autophagy in MTb-infected macrophages [61]. In murine models, pharmacological inhibition of the mammalian target of rapamycin (mTOR) pathway restores autophagic flux, enhances intracellular mycobacterial clearance and attenuates the excessive production of pro-inflammatory cytokines, thereby limiting tuberculosis-associated pulmonary tissue damage [73,74].

#### 4.1.5. Metformin

Metformin, a first-line agent in the treatment of type 2 diabetes mellitus, activates AMP-activated protein kinase (AMPK), leading to inhibition of the mTOR pathway and enhancement of autophagy in MTb-infected macrophages [67]. In vitro, metformin increases the production of mitochondrial ROS and reduces intracellular iron availability, beneficially denying MTb the essential iron it strictly requires to survive and replicate within host cells [75]. Furthermore, it promotes phagosome–lysosome fusion and suppresses the production of TNF in macrophages [76]. In retrospective human cohorts, when used in combination with standard anti-tuberculosis therapy, metformin attenuates chronic inflammation, reduces tissue-destructive immunopathology, and enhances IFN-γ secretion by CD8 and CD4 T cells [72].

#### 4.1.6. Statins

Statins, reversible inhibitors of 3-hydroxy-3-methylglutaryl-coenzyme A(HMG-CoA) reductase, exhibit pleiotropic immunomodulatory and anti-inflammatory effects in addition to their lipid-lowering properties. In vitro, by reducing cholesterol content within the phagosomal membrane of MTb-infected macrophages, statins promote phagosome maturation and autophagy, thereby enhancing mycobacterial clearance [77]. Statins have also been reported to induce phagocytic activity in murine J774 macrophages and to inhibit IFN-γ induced MHC class II expression, which is an immunomodulatory mechanism that attenuates excessive T-cell activation, thereby protecting pulmonary tissue from inflammation-induced necrosis [78]. Furthermore, statins suppress pro-inflammatory cytokines production, limit pulmonary tissue destruction and promote granuloma stability, potentially improving therapeutic outcomes [79].

#### 4.1.7. Emerging Immunomodulatory Agents

Beyond the agents discussed above, several additional compounds are under investigation in preclinical models for their potential to enhance host defense and modulate inflammation in tuberculosis. For instance, sildenafil, a phosphodiesterase-5 (PDE-5) inhibitor primarily indicated for erectile dysfunction, attenuates the immunosuppressive activity of myeloid-derived suppressor cells (MDSCs), thereby restoring protective T-cell responses against MTb [80]. Another agent is fluoxetine, an antidepressant and selective serotonin reuptake inhibitor, which interacts with macrophage serotonin receptors to increase the secretion of pro-inflammatory cytokines (IL-6, TNF-α) and induce autophagy, facilitating bacterial clearance [81]. Additionally, benztropine, traditionally used in Parkinson’s disease, antagonizes the histamine receptor 1 (HRH1) in macrophages to enhance phagosomal acidification, creating a hostile intracellular environment that limits MTb survival [82]. Another promising candidate is bevacizumab, a monoclonal antibody targeting vascular endothelial growth factor (VEGF) widely employed in oncology. Its application in TB is predicated on the structural resemblance of granulomas to solid tumors. It normalizes dysfunctional lesion vasculature, alleviating tissue hypoxia and thereby enhancing the penetration of co-administered anti-TB drugs [83].

### 4.2. Next-Generation Tuberculosis Vaccines

**Figure 3:**
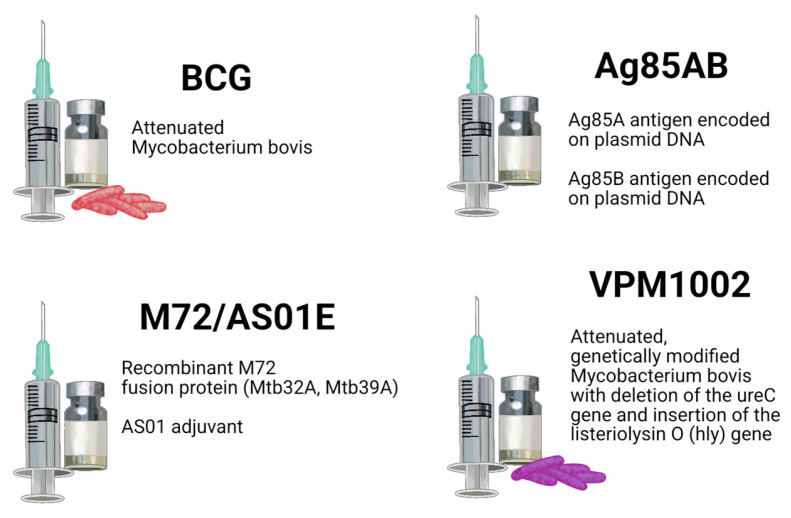
Next-generation tuberculosis vaccines next to BCG Vaccine.

BCG remains the only licensed tuberculosis vaccine. Although it confers reliable protection against tuberculous meningitis and disseminated tuberculosis in children, its efficacy against adult pulmonary tuberculosis is highly variable and generally limited [84]. Consequently, substantial efforts are ongoing to develop next-generation vaccines aimed at eliciting durable and robust cellular immune responses against MTb [85]. To illustrate recent progress, we selected three candidates that represent diverse developmental approaches: a subunit vaccine (M72/AS01E), a recombinant live vaccine (VPM1002), and a DNA vaccine (Ag85AB). We describe them below, focusing on their antigenic composition, immune response and clinical progress. They are also pictured in Figure 3.

#### 4.2.1. M72/AS01E: A Subunit Vaccine

M72/AS01E contains two MTb antigens (Mtb32A and Mtb39A) formulated with the AS01E adjuvant, which activates TLR4 and dendritic cells, thereby inducing a strong Th1 response [86,87]. MHC class II antigen presentation stimulates CD4 T cells to secrete IFN-γ, TNF-α, and IL-2, enhancing DTH, macrophage activation and granuloma stabilization [88]. Clinical trials in adults with latent tuberculosis infection have demonstrated at least three years of protection against pulmonary tuberculosis [86]. Phase III clinical trials are currently underway [89].

#### 4.2.2. VPM1002: Recombinant BCG

VPM1002 is a recombinant BCG strain engineered to express listeriolysin O, which forms phagosomal pores, facilitating partial antigen translocation into the cytosol of infected cells. This enables antigen presentation via both MHC class I (activating CD8 T cell) and MHC class II (promoting Th1 differentiation) pathways [78]. Compared with parental BCG strain, VPM1002 induces significantly enhanced CD4 and CD8 T-cell responses [90]. Phase III trials are currently underway [91].

#### 4.2.3. Ag85AB DNA Vaccine

The Ag85AB DNA vaccine encodes the MTb antigens Ag85A and Ag85B, key proteins involved in mycobacterial cell wall biosynthesis. These antigens induce a strong Th1- and Th17-type cellular immune responses, exhibit high immunogenicity, and elicit DTH and protective immunity in preclinical models. Expression of Ag85AB in host cells leads to antigen presentation via both MHC class I and II pathways, resulting in activation of CD4 and CD8 T cells and IFN-γ production [92]. However, despite encouraging preclinical results, the clinical translation of DNA-based tuberculosis vaccines remains limited and no DNA-based tuberculosis vaccines have yet advanced to human clinical trials [93].

### 4.3. Targeted Immunomodulation in Tuberculosis: Emerging Strategies and Future Directions

Effective control of pathological hypersensitivity in tuberculosis requires precise, targeted modulation of host immune responses, particularly the balance between Th1 effector responses and immunoregulatory pathways mediated by Tregs [49]. Pharmacological enhancement of Treg activity through cytokines such as TGF-β, IL-10 or IL-35 may attenuate tissue damage associated with excessive DTH, while maintaining effective control over the pathogen [94].

The PD-1/PD-L1 immune checkpoint pathway plays a pivotal role in tuberculosis by suppressing CD8 T-cell-mediated cytotoxic responses against infected M1 macrophages. Therapeutic targeting of this pathway may enhance the susceptibility of M1 macrophages to T-cell-dependent killing while sparing the relatively cytotoxity-resistant M2 macrophage population, thereby enabling more selective and controlled immunomodulation [95].

Regulation of the eicosanoid balance between prostaglandin E2 (PGE2), which stabilizes granuloma and promotes macrophage apoptosis, and leukotriene B4 (LTB4), which intensifies neutrophil recruitment and amplifies DTH responses, represents an additional promising therapeutic target. Selective pharmacological enhancement of PGE2 signaling combined with LTB4 inhibition may attenuate tissue destruction while preserving protective antimicrobial immunity [73].

Accumulating evidence supports a critical role of the gut microbiota in modulating host immune responses in tuberculosis [96]. Patients with active tuberculosis frequently exhibit profound dysbiosis, typically characterized by an altered Firmicutes-to-Bacteroidetes ratio and a marked depletion of beneficial commensal populations, including *Bifidobacterium* and *Lactobacillus* species. The reduction in these specific taxa directly decreases the systemic concentration of immunomodulatory metabolites, primarily short-chain fatty acids (SCFAs) such as butyrate and propionate. These microbial-derived SCFAs are essential for maintaining immune homeostasis, as they exert potent anti-inflammatory effects and enhance the antimicrobial capacity of macrophages against MTb [97]. Consequently, therapeutic modulation of the gut microbiota represents a promising strategy. Several interventional studies, primarily in preclinical models, have yielded positive outcomes. For instance, probiotic supplementation restores antigen presentation by lung dendritic cells, while high-fiber diets elevate systemic SCFA production to bolster respiratory defenses [98]. Furthermore, fecal transplantation (FT) effectively reverses dysbiosis, restores protective immunity, and significantly reduces the MTb burden in lungs [96]. While current evidence relies on animal models, these promising outcomes highlight the need for human clinical trials to facilitate the development of novel microbiome-targeted tuberculosis therapies.

## 5. Conclusions

Diagnosing and treating tuberculosis continues to be a key concern due to the global prevalence of the disease, the impact of diseases such as diabetes and HIV, the complexity of the immune response, and the growing problem of drug resistance. The mechanisms of MTb resistance hinder the effectiveness of antibiotics, forcing the medical community to use a limited number of available drugs. Furthermore, the development of multidrug resistance, including to isoniazid and rifampicin, which are among the frontline medications, is a serious problem. Inappropriate or ineffective treatment promotes the development of drug resistance in both MTb and other mycobacterial species, resulting in poor therapeutic outcomes.

The MT, based on type IV hypersensitivity, remains an important diagnostic tool. However, there are certain limitations, such as the possibility of false-positive and false-negative results, which significantly complicates interpretation. Alternative IGRA tests demonstrate greater specificity and are slowly becoming the preferred method for diagnosing latent infection. Approaches intended to adjust the host immune reaction are gaining importance in treatment, aiming to both strengthen defense mechanisms against mycobacteria and limit destructive inflammation. Coordinated efforts based on the use of modern diagnostic tools, immunomodulation, and new vaccines allow us to optimistically look to the future towards reducing morbidity and mortality from tuberculosis.

## Figures and Tables

**Table 1:** The DTH mediators and their role in tuberculosis.

DTH Mediator	Main Cellular Source Direction of Effect on DTH	Direction of Effect on DTH	Role in Tuberculosis	Key References
IFN-γ	Th1 cells, CD8^+^ T cells, NK cells	↑ Activation	Causes activation of macrophages, increases intracellular killing of tuberculosis bacilli	[39]
TNF-α	Macrophages, T cells	↑ Activation	It acts synergistically with interferon gamma and is responsible for the formation and maintenance of granulomas.	[40]
TGF-β1	Treg, macrophages	↓ Suppressive	Inhibits Th1 cell function and IFN-γ production, which increases bacterial counts.	[41]
IL-10	Treg cells, M2 macrophages	↓ activation	Limits excessive inflammation and tissue damage but may impair intercellular killing of *M. tuberculosis*	[42]
Treg	Natural and induced CD4^+^CD25^+^FoxP3^+^ T cells	↓ Activation	Controls tissue damage but may reduce protective anti-mycobacterial immunity in active TB	[43,44]
Eicosanoids (PGE_2_, LTB_4_)	Macrophages, neutrophils	Modulatory (context-depends) PGE_2_ ↓/Regulatory LTB_4_ ↑ Activation	Balance between PGE_2_ and LTB_4_ determines apoptosis vs. necrosis of infected macrophages and influences disease progression	[45]

**Table 2:** The interpretation of MT and determination of reactive hypersensitivity to PPD.

Localized Hardening ≥ 5 mm	Localized Hardening ≥ 10 mm	Localized Hardening ≥ 15 mm
HIV-infected individuals	Recent arrivals (less than 5 years) from high-prevalence countries	No known risk factors for tuberculosis
Current contacts with people infected with tuberculosis	Drug injections	
Nodular or fibrotic changes on chest radiograph consistent with old tuberculosis	People who work in high-risk congregate settings such as prisons, hospitals, facilities for AIDS patients, and mycobacteriology laboratories	
Patients who have been taking systemic corticosteroids for more than six weeks, or who are on a daily dose of prednisone of 15 mg or more or an equivalent	High-risk patients diagnosed with diabetes, leukemia, chronic renal failure, low body weight, etc.	
Organ transplant recipients and other immunosuppressed patients	Infants, children, adolescents exposed to adults in high-risk categories	

## Data Availability

No new data were created or analyzed in this study. Data sharing is not applicable to this article.
